# Acute blood flow restricted exercise to treat Duchenne muscular dystrophy: would it be efficacious?

**DOI:** 10.3389/fphys.2013.00114

**Published:** 2013-05-16

**Authors:** Jeremy P. Loenneke, Robert S. Thiebaud, Takashi Abe, Igor G. Manfro, Pedro J. Marin

**Affiliations:** ^1^Neuromuscular Research Laboratory, Department of Health and Exercise Science, The University of OklahomaNorman, OK, USA; ^2^Department of Kinesiology, Indiana UniversityBloomington, IN, USA; ^3^Bone Research Laboratory, Department of Health and Exercise Science, The University of OklahomaNorman, OK, USA; ^4^Research Centre on Physical Disability, ASPAYM Castilla y León FoundationValladolid, Spain; ^5^Laboraotry of Physiology, European University Miguel de CervantesValladolid, Spain

The most common form of muscular dystrophy is Duchenne muscular dystrophy (DMD), which is an X-linked disorder affecting 1 in 3500 newborn males across the world. DMD is caused by the lack of dystrophin which is a protein that provides stability to the sarcolemma and is likely involved in the transmission of force between the extracellular matrix and the intracellcular contractile apparatus (Lovering and Brooks, 2013). Due to the absence of this protein, the sarcolemma is easily damaged in response to muscle contraction (Markert et al., 2012). Although there is no cure for DMD, exercise has been proposed as a possible treatment; however, the risk of muscle damage is a large concern for this population (Markert et al., 2012). Therefore, traditional strength training which includes high load eccentric contractions may need to be avoided in patients with DMD due to the susceptibility of muscle damage with lengthening contractions. In addition, although submaximal exercise may exert some benefits for this population, the low load exercise is unlikely to be an optimal stimulus for maintaining or increasing muscle function. Interestingly, there are numerous studies (>40) in healthy subjects which suggest that submaximal exercise in combination with blood flow restriction (BFR) can elicit muscle adaptations similar to that observed with higher load resistance training (Loenneke et al., 2012c) without increasing indices of muscle damage (Loenneke et al., 2011).

Briefly, BFR is a stimulus commonly applied with specialized pressure cuffs placed at the top of a limb which are inflated to a set pressure throughout exercise. The pressure applied should be high enough to occlude venous return from the muscle but low enough to maintain arterial inflow into the muscle. Available evidence indicates that the pressure applied should be based on the size of the limb (i.e., bigger the limb higher the pressure) (Loenneke et al., 2012b). The proposed mechanisms (Loenneke et al., 2012a) behind the effects of low load exercise in combination with BFR on skeletal muscle include acute muscle cell swelling, increased fiber type recruitment from metabolic accumulation, decreased myostatin (48 h after last training session), decreased atrogenes (8 h post exercise), and the proliferation of satellite cells (8 days into training intervention, 3 and 10 days after cessation of training) (Nielsen et al., 2012).

A recent review discussed 5 mechanisms of DMD pathology which may improve or worsen as a result of exercise training which included: (1) mechanical weakening of the sarcolemma; (2) inappropriate calcium influx; (3) aberrant cell signaling (angiogenesis); (4) increased oxidative stress; and (5) recurrent muscle ischemia (Markert et al., 2012). The purpose of this manuscript is to discuss each one of these mechanisms as it relates to what is known about low load resistance exercise in combination with BFR.

## Mechanical weakening

Muscles that lack dystrophin result in sarcolemmal fragility, making the muscle easily susceptible to damage (Menke and Jockusch, 1995). Maximal eccentric contractions are known to result in muscle damage; however, this is not observed with submaximal exercise in combination with BFR (unpublished observations). Despite this lack of muscle damage, pronounced changes in muscle mass and strength occur (Loenneke et al., 2012c). Recent research suggests that the concentric muscle action is playing the most important role with respect to changes in muscle mass and strength (Yasuda et al., 2012). Therefore, it is conceivable that patients with DMD could possibly increase muscle size and strength from completing submaximal concentric only muscle actions with BFR, which has not been observed to increase any indices of muscle damage in healthy subjects (unpublished observations). However, it is acknowledged that this response may be different in patients in DMD, who already have a fragile sarcolemma.

## Calcium influx

The maintenance of calcium at an appropriate level in skeletal muscle (resting cytosolic concentration of ~50 nM) is important and when calcium is not properly regulated, muscle degradation can occur. The mechanism behind this muscle degradation is not completely known, however, one proposed mechanism is an increase in calpains. Calpain 3 is tightly bound to titin and is involved in degradation by disassembling the outer layers of proteins from the myofibril. Muscles of mdx mice have increased calpain concentration and activity (Spencer et al., 1995), which is diminished in mdx mice that overexpress calpastatin (Spencer and Mellgren, 2002). Interestingly, it has been recently hypothesized that calpastatins are likely increased with the application of BFR (Loenneke et al., 2012d). The possible increase in calpastatin with BFR is thought to occur through signaling of the beta 2 adrenoceptor following binding of norepinephrine. Although theoretically plausible due to the increase in norepinephrine following the application of BFR, future work is needed to determine if the increase in calpastatin actually occurs in healthy or DMD muscle.

## Angiogenesis

Angiogenesis in skeletal muscle results from hypoxia, shear stress, and increases in growth factors such as vascular endothelial growth factor (VEGF) (Larkin et al., 2012). In addition, there is evidence that the frequency of mesenchymal stem cells (MSCs) correlates with blood vessel density (Da Silva Meirelles et al., 2009) which suggests that angiogenesis may increase the availability of MSCs for regenerating tissue in patients with DMD. Recently, low load resistance exercise in combination with BFR has been observed to increase post-exercise expression of mRNA related to skeletal muscle angiogenesis in healthy young adults (Larkin et al., 2012). This provides proof of concept for possible future work in patients with DMD to determine if tissue regeneration is possible following exercise in combination with BFR. However, it should also be mentioned that if the availability of MSCs is increased with exercise in combination with BFR, these MSCs would carry the same genetic mutations as the patients' somatic cells (Markert et al., 2012).

## Oxidative stress

Oxidative stress is a biological phenomenon marked by an imbalance between reactive free radicals and antioxidant defenses. When oxidative stress is severe and/or prolonged, the natural defense system can be overwhelmed, leading to subsequent oxidative damage of lipids, proteins, and DNA. Lipids comprise part of the sarcolemma and these lipids are preferentially attacked by reactive oxygen and nitrogen species (Murphy and Kehrer, 1989). If this attack is not corrected by antioxidants, it could lead to the destruction of the contractile units of the muscle cell, actin, and myosin. Although heavy resistance training can increase oxidative stress, submaximal exercise with BFR has not been shown to increase oxidative stress in those who are healthy (Goldfarb et al., 2008) or in those with heart disease (Madarame et al., 2013). Therefore, exercise with BFR may be able to increase/maintain muscle function in those with DMD without further increasing levels of oxidative stress.

## Recurrent muscle ischemia

There is some mechanistic evidence for recurrent muscle ischemia in the muscle of DMD patients. Briefly, studies have shown that in healthy skeletal muscle, blood flow during exercise is increased to the working muscle due to nitric oxide release. However, this does not occur in DMD muscle, therefore the reflex sympathetic vasoconstriction that accompanies contraction of DMD muscle is unopposed by nitric oxide mediated vasodilation resulting in ischemic muscle (Markert et al., 2012). Although the application of BFR during exercise is acute and brief, it is possible that this impaired vascular control in those with DMD could contraindicate them to this mode of exercise. The only evidence available to suggest BFR in combination with exercise may be safe in this population comes from a single case study from a patient with idiopathic inflammatory myopathy (Gualano et al., 2010). It has been hypothesized that idiopathic inflammatory myopathies also have impaired vascular function (Grundtman and Lundberg, 2009), suggesting that improvements in muscle function may occur following acute bouts of BFR exercise in patients with a compromised vascular system. However, the mechanism of vascular dysfunction for idiopathic inflammatory myopathy is different than that for DMD; therefore direct comparisons between the two conditions should be made with caution.

## Conclusion

In conclusion, we wish to suggest the possibility that the application of BFR in combination with exercise may be beneficial for maintaining/increasing muscle function in patients with DMD. This is based on the observations that BFR in combination with submaximal exercise increases muscle function without increasing markers of muscle damage or oxidative stress. Future research is needed to confirm that these effects largely observed in healthy subjects, would transfer to patients with DMD. It may be useful to first design and test this theory in an animal model to determine proof of concept.
